# Prevalence of familial multiple sclerosis in Iran: A systematic review and meta-analysis

**Published:** 2017-04-04

**Authors:** Mahmood Moosazadeh, Ravanbakhsh Esmaeili, Mohammad Mehdi Nasehi, Ghasem Abedi, Mahdi Afshari, Fereshteh Farshidi, Motahareh Kheradmand

**Affiliations:** 1 Health Sciences Research Center, School of Health, Mazandaran University of Medical Sciences, Sari, Iran; 2 Department of Medical-Surgical Nursing, Nasibeh School of Nursing and Midwifery, Mazandaran University of Medical Sciences, Sari, Iran; 3 Department of Pediatric Neurology, School of Medicine, Mofid Hospital, Shahid Beheshti University of Medical Sciences, Tehran, Iran; 4 Department of Community Medicine, School of Medicine, Zabol University of Medical Sciences, Zabol, Iran; 5 Health Sciences Research Center, School of Health, Mazandaran University of Medical Sciences, Sari, Iran

**Keywords:** Familial, Multiple Sclerosis, Meta-Analysis, Prevalence, Iran

## Abstract

**B** **ackground:** Familial history of multiple sclerosis (MS) has been considered as one of the etiologic factors of MS by several studies. It is valuable to combine the results of these studies. The aim of this study is to estimate the pooled prevalence of familial MS in Iran using meta-analysis.

**Methods:** Using relevant keywords, national and international databanks were searched. Considering the significant heterogeneity between the results, random effect model was utilized to estimate the pooled prevalence of familial MS using Stata software.

**Results:** After screening the selected articles, 15 studies with total sample size of 6248 (from 60 to 1718) were identified eligible for final meta-analysis. Overall prevalence of familial MS in Iran was estimated as of 11.4% [95% confidence interval (CI): 8.7-14.1]. Point prevalence varied between 3.3% and 26.7%.

**Conclusion:** Our study showed that the familial prevalence of MS among Iranian people is relatively high. More studies are warranted to investigate the effect of familial history as a risk factor for MS.

## Introduction

Multiple sclerosis (MS) is an inflammatory disease which is characterized by multifocal inflammation, demyelination, gliosis, and neuronal loss of the brain and spinal cord.

In the United States, 400000 people are affected by MS and this inflammatory disease affects 2.5 million individuals worldwide, the prevalence of MS varying greatly with geography.^1^ It is hypothesized that MS is a complex and multifactorial disease and is believed several environmental factors and genetic risk factors, as well as their interaction, are the causes of disease.^2^^,^^3^ Some of nongentical risk factors of MS are included lack of sunlight exposure (mediating vitamin D synthesis), exposure to infectious agents, smoking, immunization, hormonal factors, nutritional habits, and psychological stress.^3^ Inheritance of MS does not follow Mendelian model. Multiple independent or interacting polymorphism genes with small or moderate effect have role in etiology of MS.^4^

Regardless of sporadic occurrence of MS, a considerable proportion of patients (almost 20%) are related by family.^5^ The greater recurrence risk of MS in twins, sibling, conjugal MS individuals, and lower recurrence risk in adoptees has clearly demonstrated an important role of genetic factors in etiology of disease and familial aggregation of disease.^6^^-^^8^ It also strongly supports that MS susceptibility is polygenic.^9^

It is well known that MS accumulate within families. The degree of the familial risk^5^ and its difference in various geographic remain uncertain.^10^

Although in 15 years ago Iran was considered to be located in a low-frequency zone of MS (prevalence rate fewer than 5 per 100000),^11^ recent epidemiological studies reported the different prevalence of MS in different provinces in Iran. Its prevalence ranged from 7.4 to 89 per 100000 with an average prevalence and incidence of 54.51 and 5.87 per 100000 people, respectively (Isfahan^12^ and Mazandaran).^12^^-^^14^ Despite the partially high prevalence of MS in Iran to our knowledge, there is no systematic review regarding the prevalence of familial MS in Iran. The aim of this study was identifying the prevalence of familial MS using a systematic approach.

## Materials and Methods

We searched electronic papers published from 2000 to 30 August 2015. The search was conducted using international databanks including Web of Sciences, PubMed, Google Scholar, and Scopus. National databanks such as SID, Iranmedex, Magiran, and Irandoc were investigated with appropriate keywords. The following keywords and their Farsi equivalents were applied for electronic search: “Multiple sclerosis or MS AND familial (OR family) AND prevalence, prevalence, frequency, familial, Iran, epidemiology.”

Two independent researchers conducted the search during 1-15 September 2015. They also investigated all references of the articles to increase the search sensitivity. A third researcher randomly evaluated the results to identify any probable ignored article. Moreover, none electronic papers were investigated by the research team. To find relevant gray literatures, the experts of some research centers were interviewed. 

We extracted full texts or abstracts of all articles identified during the primary search. Then, duplicates were excluded from the study. Finally, none relevant studies (by reviewing the titles, abstracts, and full texts, respectively) were removed. To minimize re-print bias, we had to investigate the results of each study and omit repeated articles. 

Selected papers were quality assessed using a previously applied checklist.^13^ The quality of studies was assessed using checklist designed according to the Strengthening the Reporting of Observational Studies in Epidemiology (STROBE) contents^14^ which included 22 questions about regarding different aspects such as sample size and sampling methods, study design and study population, methods and instruments for data collection, variable definition, statistical methods, study objectives and illustration of the results. Each study was assigned a score from 0 to 44 then all studies were categorized into low quality, moderate quality, and high quality. We excluded studies with low quality from the final analysis.^14^

Title, first author name, date of study conduction, type of the study, sample size and sampling methodology, language of the article and prevalence of familial MS were extracted from each study. The information was entered into the Excel spreadsheet.

All Persian and English-written papers reporting sample size and prevalence of familial MS and achieved enough quality scores were included in the meta-analysis. 

Studies did not report the familial MS prevalence, those with unknown sample size, abstracts presented in congresses without full text, case reports, and case–control studies as well as clinical trials and finally, studies with low-quality scores were excluded from the meta-analysis.

Stata SE (version 11, Stata Corporation, College Station, TX, USA) software was utilized for statistical analysis. The standard error of the MS prevalence was estimated based on binomial distribution formula. According to the amounts of the heterogeneity indices [Cochrane (Q) test and I-square index)] fixed or random effect models were applied to combine the prevalence. In addition, studies with the most influence on the heterogeneity were determined using sensitivity analysis. Factors associated with the heterogeneity were investigated using meta-regression models. We also illustrated the point and pooled prevalence of MS by forest plots. The weight of each study was shown by the sizes of the boxes and bilateral lines indicating the 95% confidence interval (CI) of the prevalence.

**Figure 1:**
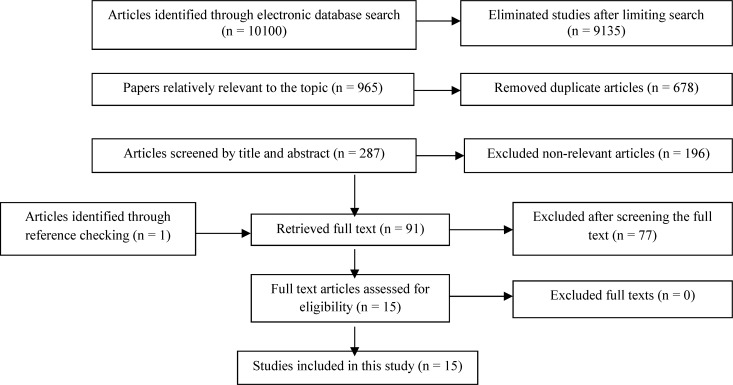
Literature search and review flowchart for selection of primary studies

## Results

The primary search identified 10100 articles which were reduced to 287 studies after restricting the search strategy as well as duplicates exclusion. Reviewing titles and abstracts revealed 196 irrelevant papers. Investigating the full texts showed 77 none eligible studies and reference review added one relevant study to the results. Finally, 15 articles were entered into the meta-analysis (Figure 1). 

Studies had been published from 2003 to 2015 and written in Persian (six studies) and English (eight studies). Results of an unpublished study were received from the authors. These studies were conducted in different provinces such as Isfahan (two papers), Tehran (four papers), Razavi Khorasan (one paper), East Azerbaijan (one paper), Kermanshah (one paper), Hamadan (two papers), Zanjan (one paper), Qom (one paper), Mazandaran (one paper), and multiple provinces (one paper). In total, these studies had been conducted among 6248 individuals differed from 60 to 1718 (Table 1).

**Table 1:** Characteristics of the primary studies included to this meta-analysis

**References**	**Local study**	**Publication ** **language**	**Publication ** **year**	**Sample size**	**Prevalence**
Ashtari, et al.^15^	Isfahan	Persian	2011	593	20.1
Baghizadeh, et al.^16^	Tehran	English	2013	338	13.6
Danesh-Sani, et al.^17^	Khorasan-Razavi	English	2013	500	15.4
Ghabaae, et al.^18^	Tehran	English	2007	70	8.6
Hashemilar, et al.^11^	East Azarbaijan	English	2011	1000	7.1
Mazaheri, et al.^19^	Hamadan	Persian	2007	155	12.9
Payamani and Miri^20^	Tehran	Persian	2011	200	13.0
Pourmemari, et al.^21^	Zanjan	Persian	2011	96	7.3
Rezaali, et al.^22^	Qom	English	2013	592	11.2
Rezaie and Panahi^23^	Hamadan	Persian	2005	60	26.7
Saadatnia , et al.^24^	Isfahan	English	2007	1718	12.2
Taraghi, et al.^25^	Mazandaran	Persian	2007	101	7.0
Kalanie, et al.^26^	Tehran	English	2003	200	5.0
Nasehi, et al.^27^	Some province	English	2015	177	16.4
Saman-Nezhad, et al.^28^	Kermanshah	English	2013	448	3.3

**Figure 2:**
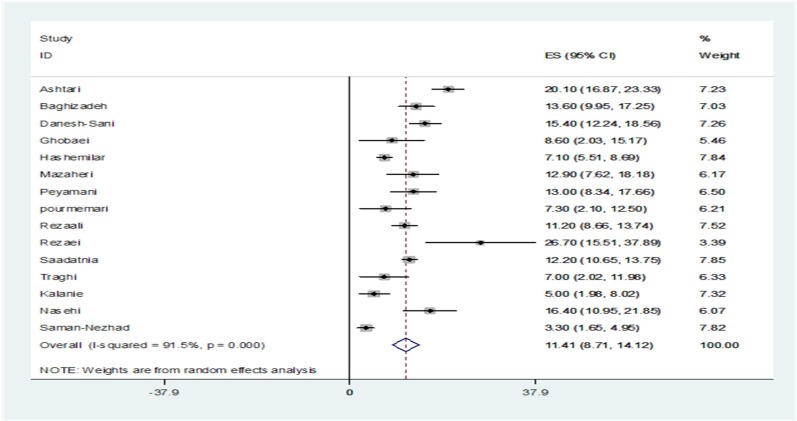
The prevalence of familial multiple sclerosis (MS) in among primary studies and prevalence of pooled estimate in Iran

The prevalence of familial MS was reported as 3.3% in Saman-Nezhad, et al. study, among 448 persons in Kermanshah^28^ to 26.7% in Rezaie and Panahi study, among 60 citizens in Qom^23^ (Table 1). Significant heterogeneity was observed between the results of the primary studies (Q = 165.02, I^2^: 91.5%, P < 0.001). Therefore, random effect model was used to combine the results. The total prevalence of familial MS among Iranian people was estimated as of 11.4% (95% CI: 8.7-14.1) (Figure 2).

According to the meta-regression models, geographical area was not associated with MS prevalence (β = 0.02; P = 0.900). We did not conduct subgroup analysis based on the geographical areas due to the low number of the studies.

During the primary and secondary steps of the sensitivity analysis, Saman-Nezhad, et al.^28^ and Hashemilar, et al.^11^ studies were found as studies with extreme results. Although excluding these studies from the meta-analysis changed the I^2^ (86.7% and 82.2% respectively), the heterogeneity was still remained.

## Discussion

Results of our study showed the prevalence of familial MS was in the range of 3.3-26.7%, and the pooled prevalence was estimated about 11.0% for Iran.

Nielsen, et al.^5^ utilized a nationwide registry data and reported that risk of MS in families with a positive history of MS was 7 times more than normal population (relative risk = 7.1, 95% CI: 5.8-8.8). They also reported that first-degree relatives had 2.5% (95% CI: 2.0-3.2) excess risk irrespective of their gender and the relative. Carton, et al.^8^ also reported 10-fold to 12 increased risk of MS for first degree, 3-fold for the second degree relatives increase risk for the risk for the first degree in their population (674 probands with MS in Flanders).

In a study in Jordan, the prevalence of family history of MS reported in 9.4% of the patients.^29^

In study that was carried out by Fricska-Nagy, et al. in Hungry, the familial prevalence of MS are estimated between 5% and 10%.^30^

In favor of our study results, the finding of population-base cohort study showed that prevalence of MS among first-degree and non-biological relatives of patients with MS was not more than general population but it was significantly less than biological relatives. These findings indicate the role of genetics factors in familial aggregation of MS. They did not detect any effect of shared environment was detectable.^9^ These findings demonstrate the familial clustering of the disease. Studies have shown factors influencing onset age also increase recurrence risk in patients’ siblings. This can support the opinion that individuals with a greater genetically influenced susceptibility to MS tend to have an earlier onset, which means genes influencing susceptibility also contribute to determining precocious disease.^6^

Most etiologic studies focused mainly on the environmental factors and rarely investigated the genetic causes. Some researchers believe that environmental factors provide background of the disease, while genetic factors exacerbate the environmental effects. Risk of developing MS is increased more than 2-4% among first-degree relatives. The corresponding risk for other family members was 0.1%. Another study conducted in 2007 rejected the monogenic cause of MS and reported that two genes are responsible for developing the disease.^7^^,^^31^

The above results show the stronger association of the genetic factors with MS. Our meta-analysis provided descriptive evidence regarding MS prevalence. However, analytic studies are required to prove the causal inference. 

Unfortunately, we could not estimate the pooled prevalence based on different genders and geographical areas because of inadequate relevant studies. Considerable heterogeneities between the results of the primary studies was another limitation of the current study. Geographical area might be one of the related factors for this heterogeneity which was not investigated due to lack of enough information.

## Conclusion

Our findings showed that familial prevalence of MS among Iranian people is relatively high. Further studies are suggested to investigate the effect of familial history as a risk factor for MS. This finding support the involvement of genetic factors in the etiology of MS and consulting with geneticist for individuals with a positive history of MS must take into account.

## Notes:

**How to cite this article:** Moosazadeh M, Esmaeili R, Nasehi MM, Abedi G, Afshari M, Farshidi F, et al. Prevalence of familial multiple sclerosis in Iran: A systematic review and meta-analysis. Iran J Neurol 2017; 16(2): 90-5.
